# Suppressive, Curative, and Prophylactic Effects of *Maesa lanceolata* Forssk. against Rodent Malaria Parasite *Plasmodium berghei*

**DOI:** 10.1155/2022/8901555

**Published:** 2022-11-12

**Authors:** Eyob Tekalign, Getnet Tadege, Nebeyi Fisseha, Dejen Nureye

**Affiliations:** ^1^Department of Medical Laboratory Sciences, College of Medicine and Health Sciences, Mizan-Tepi University, P.O. Box 260, Mizan-Aman, Ethiopia; ^2^Department of Pharmacy, School of Pharmacy, College of Medicine and Health Sciences, Mizan-Tepi University, Mizan-Aman, Ethiopia

## Abstract

The artemisinin partial resistance is believed to be spread to artemisinin-based combination therapy partner drugs. As a result, new antiplasmodial compounds are required to treat resistant malaria infections. In the invention of antimalarial substances, claimed medical plants are precious resources. So, the current study was designed to assess the antiplasmodial effects of *Maesa lanceolata* in mice. In this study, preliminary phytoconstituent and *in vivo* acute oral toxicity tests were done. Early infection, established infection, and residual infection tests were employed to determine the antimalarial effects of the test drugs. Three doses (200, 400, and 600 mg/kg) of the extracts were provided orally to the test mice. Analysis of variance (one-way) followed by post hoc Tukey's test was used to analyze the difference between and within groups. Terpenoids, tannins, saponins, flavonoids, and alkaloids were detected in the phytochemical constituent analysis. Both 80% methanolic crude extract and solvent fractions had no toxic result at the 2000 mg/kg dose. All test drug doses suppressed parasite levels in a significant manner at all tests. The activity of chloroform fraction (maximum percentage suppression, 81.28%) overwhelms the crude extract activity. The curative effects of 80% methanolic crude extract, with a maximum of 80.22% parasitemia suppression, were greater than its suppressive and prophylactic effects. The 400 mg/kg dose of chloroform fraction resulted in a maximum survival period (18 days) than other doses of tested materials. The results of this investigation provide support for the activity of *M. lanceolata* leaf extract against malaria.

## 1. Introduction

Malaria is a vector-borne infection caused by parasites that infect erythrocytes. It is transmitted by the *Plasmodium* carrier female *Anopheles* mosquito. The most common malaria symptom is fever. In severe cases, malaria may result in loss of consciousness, convulsions, breathing difficulty, severe anemia, and multiple organ failure [1]. Changes in land use have increasingly been escalating all over the earth in the past decades. This phenomenon can have sound effects on the spatial and temporal distribution of vector-borne infections including malaria through changes in ecology and habitat. Nowadays, malaria accounts for 2.6% of the total global disease burden [2]. According to the World Health Organization (WHO), there were over 241,000,000 malaria cases in 2020, of which 228,000,000 were reported from the African region. Around 627,000 malaria deaths were also reported globally this year. Compared to the 2019 report, the morbidity and mortality due to malaria have increased in 2020. This disease (preventable and treatable infection) continues to pose a shocking impact on the world's population [3, 4]. Malaria-related illness and death cause significant private costs and societal costs in many tropical countries [5].

About 10% of the overall disease burden in Africa is caused by malaria. In Africa, pregnant mothers and young kids continue to suffer from the curse of malaria. It is also a primary cause of mortality among African children, causing around 20% of all deaths of less than five years old children. Moreover, malaria is still among the prioritized community health problems in Ethiopia [2]. It is present in almost 70% of Ethiopia, with 52% population at risk. Malaria transmission is very much seasonal and differs geographically across the nation [6]. It is the most common cause of in-patient admission (13-26%) and out-patient visitors (10-40%) at the country level with 13–35% resultant death rates. It is also one of the major causes of malaria cases and deaths among Ethiopian adult populations [2].

Out of the four hundred different species, around 60 species of mosquito transmit malaria to humans. In Africa, *Anopheles gambiae* species have been considered the most dangerous and common in rural areas. Since the majority of African *Anopheles* mosquitoes breed in rural settings, malaria is traditionally known as a rural disease. However, *Anopheles stephensi*, the main malaria mosquito in India, has currently been identified in cities and towns in urban locations in Djibouti, Ethiopia, and Sudan. Hence, malaria can become an escalating problem in urban Ethiopia and somewhere else in Africa [7–9].

The emergence of *A. stephensi*, the invasive malaria vector, is a serious risk to the global health effort in Africa. It is a proficient vector of *P. falciparum* and *P. vivax* and a likely vector of zoonotic Plasmodium species. According to the World malaria report, the spread of *A. stephensi* due to zoophilic, exophagic, and exophilic preferences is an additional threat to malaria control and prevention in Africa. It has shown resistance to insecticides (insecticide-treated nets and indoor residual spraying), delaying the control of *A. stephensi* via these methods. Moreover, this vector usually breeds in manmade water storage areas, which in turn means an extended period of malaria transmission because a lot of people store water secondary to a lack of sustainable water access. This concern is predominantly vital in urban and periurban places, where there is a crowded population, poor health-care and vector control systems, and the continual need to stock up water. This means that malaria epidemics are very much expected in those settings [10]. These circumstances introduced an extra obstacle in halting malaria spread and create challenges in combating malaria.

As compared to the condition before the year 2000, the number of malaria-infected people has decreased, especially in South America and sub-Saharan Africa. Disappointingly, malaria nowadays appears to be on the rise in various places where it was formerly under control [7]. Immunization with the four doses of Mosquirix® (RTS, S vaccine) brings only a 30% decrease in malaria mortality in children. Besides, the malaria vaccine is not available for adults [1]. The artemisinin partial resistance is expected to spread resistance to artemisinin-based combination therapy (ACT) partner drugs. This intimidation emphasizes the necessity for a robust antiplasmodial agent's pipeline attributing compounds with new mechanisms of action. There is also a fear that the same could occur in the African continent. Of course, there is an emergence of resistance against antimalarial agents in East Africa. While we await novel compounds from the new drug development pipeline (seven of which are in translational and human exploratory studies), they might fail at later stages of the pipeline [3, 11]. As a result, searching for new antiplasmodial compounds should be a continuous activity to treat resistant malaria infections. In the discovery of novel antimalarials, medicinal plants are valuable resources. Using them in the past has led to the discovery of plant-based malaria treatments that have saved millions of lives. The antimalarial drugs artemisinin and quinine are obtained from natural products, *Artemisia annua* and Cinchona bark, respectively [12].

The Myrsinaceae family is composed of 1,000 species in 33 genera including the genus *Maesa*. It is characterized by its contents 2,5-dihydroxy-3-alkyl benzoquinones and various triterpenoids based on ursane and/or oleanane skeleton [13]. Phytoconstituent analysis performed on different *Maesa* species resulted in the detection of a variety of terpenoid saponins. For example, six related triterpenoid saponins having activity against leishmaniasis were isolated from *M. balansae* [14]. Three triterpenoid saponins maetenosides [13], maelexins [15], and maejaposides [16] are present in *M. tenra*, *M. laxiflora*, and *M. japonica*, respectively. A novel phenolic compound, which is known as maesol, was isolated from both *M. montana* and *M. indica* seeds [17].

**Figure 1:**
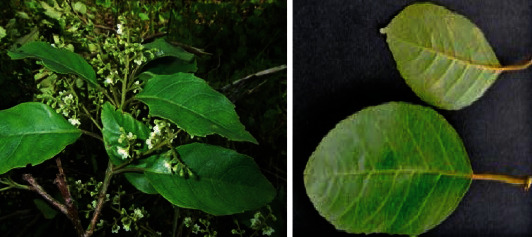
Pictures of *Maesa lanceolata* Forssk. leaves.

*Maesa lanceolata* Forssk. is one of the Myrsinaceae families and is commonly found in South Africa, Madagascar, Arabia, and tropical Africa including Ethiopia. It is a 2 to 20 m tall tree or shrub that grows either near the periphery of an evergreen forest or in open woodland and valleys with an altitude of 1,350 to 3,000 m [18]. It is called Kelewa in Amharic [19], Abbayyii in Afaan Oromo [20], and Gowacho in Sidama [21]. About 10 triterpenoid saponins (acylated) were obtained from the leaves (Figure 1) of this plant [22, 23]. Two benzoquinone derivatives (lanciaquinone and 2,5-dihydroxy-3-[nonadec-14-enyl]-benzoquinone) were also extracted from *M. lanceolata* fruits [13]. Myrcene was identified in the chloroform leaf extract of Ethiopian *M. lanceolata* [24].

There are several reports in the journals on the bioactivities of *M. lanceolata*. The seeds of the plant have application as a vermifuge. The oil obtained from its seeds is useful for lubricating the baking plate of bread and Injera [18]. The dihydromaesanin, maesanin, maesanin di-ethylether, and isomeric mixtures of benzoquinones showed cytotoxic effects. The isomeric acylated benzoquinones produced significant antioxidant and antiproliferative activity [25]. The methanol extract exhibited virucidal action against herpes simplex and vesicular stomatitis viruses. Related to this effect, maesasaponin mixture A was detected to be an active principle [26]. Similarly, in another study, maesasaponin mixture B (a virucidal saponin mixture containing 6 homologous oleanane-type triterpenoid saponins) was obtained from this extract [27]. In addition to the virucidal action [28], triterpenoid saponins have antimutagenic, molluscicidal, and hemolytic activity [27, 29, 30]. Extracts of *M. lanceolata* were also shown *in vitro* fungistatic activity against pathogenic fungus species affecting plants [31]. Maesanin was also found to have an inhibitory effect on the 5-lipoxygenase enzyme, thus, indicating the anti-inflammatory activity of the plant [32].

Water extracts of *M. lanceolata* root and stem barks were very active against *Cryptococcus glabrata.* On the other hand, water and methanolic extracts from its root were effective against *Shigella dysenteriae*, *Escherichia coli*, *Staphylococcus aureus*, *Pseudomonas aeruginosa*, and *C. neoformans* [33, 34]. The *in vitro* cytotoxicity experiment showed that the majority of the extracts from *M. lanceolata* were relatively nontoxic. Ethnopharmacological studies also reported that *M. lanceolata* has activity against *Vibrio cholera* and *Salmonella typhi* [34]. The acetone extract of *M. lanceolata* leaves showed a good safety profile, excellent *in vitro* antibacterial activity, and relatively low antioxidant activity [35, 36]. An investigational work has shown that the hydroethanolic leaf extract of *M. lanceolata* alleviates the manifestations of chemical-induced ulcerative colitis in laboratory rats [37]. Research done in East Africa revealed that the chloroform extract of *M. lanceolata* leaves showed *in vitro* antiplasmodial activity [38]. The 80% methanolic leaf extract also displayed antiplasmodial activity against chloroquine-resistant *P. falciparum* strains [39]. Moreover, the hydromethanolic leaf extract of *M. lanceolata* and its fractions had *in vitro* antimicrobial activities against bacteria and fungi [18]. Quercitrin isolated from Ethiopian *M. lanceolata* seeds has shown noteworthy anticancer activity [40].

Ethnobotanically, the plant *M. lanceolata* is utilized in the local communities of Africa to prevent and treat various illnesses. It is used to treat dysentery, hypertension, and dermatosis elsewhere in Africa [37]. In Kenya, seeds and ground fruit are used in mixed form as an anthelminthic agent; seeds and coarsely powdered fruit are indicated for backache; seeds and fruit extracted from boiled water are used for malaria treatment; seeds and dried fruit are used to stimulate appetite; decoction of the root is applied for reduced body strength and as a nutrient; seeds and grounded fruit soaked in water are used for syphilis and gonorrhea [40]. In Kenya and Tanzania, the root and stem barks of *M. lanceolata* prepared in decoction form are used to treat malaria. Its leaf is also used to manage malaria in Tanzania [39, 41, 42].

In Ethiopia, the fruits and seeds of this plant are crushed and mixed with water to take orally to treat ascariasis [19]. Its bark and leaf are used to control diarrhea [21]. A combination of dried/fresh leaf of *M. lanceolata* and *Clematis simensis* leaf powder mixed with butter is applied topically in treating leprosy [43]. Crushed fresh leaves rubbed on the body are used to treat external parasites [20]. The leaf and fruit parts of *M. lanceolata* are used as antimalarial, and its fruit juice sprayed in the house is used as an insecticidal agent by local communities [44, 45]. Taking the past studies into consideration, this study was undertaken to investigate the antimalarial effects of the leaf extract of *M. lanceolata* against *Plasmodium berghei* in experimental animals and can help to confirm its traditional claimed use by traditional practitioners.

## 2. Materials and Methods

### 2.1. Plant Sample Collection

In October 2020, fresh leaves were collected from the area called Andracha Woreda, Sheka Zone, South West of Ethiopia which was 576 km far from Central Ethiopia. At the time of transportation, the leaves were covered in a plastic sheet. The harvested plant was recognized and authenticated as *M. lanceolata* Forssk. by a taxonomist working in the National Herbarium, College of Natural and Computational Sciences, Addis Ababa University. The sample voucher (No. ET 001/2020) has been kept there for future reference.

### 2.2. Laboratory Animals and Inoculum Plasmodium

The study mice were acquired from the national organization known as Ethiopian Public Health Institute (EPHI). Six to eight weeks old healthy albino male mice with 23-33 g body weight were used for the antimalarial test. Female mice with the same range of age and body weight were used for the acute toxicity test because they are more sensitive than male ones [46]. They were placed in the plastic cages at normal room temperature and 40-50% relative humidity with a 12 h light-dark cycle. Drinkable water and a standard pellet diet were provided for them. The study mice were acclimatized for 3 to 5 days before the experiment. ANKA strain *P. berghei* (a chloroquine-sensitive) was received from the EPHI and maintained weekly by serial passage of infected blood from infected to healthy mice. The handling and caring of the laboratory mice were according to the rules described in [47]. The detail of the procedures was reviewed and accepted by the Research and Ethical Committee of the School of Pharmacy, Mizan-Tepi University with approval number (SOP6/11/13).

### 2.3. Crude Extract and Solvent Fractions

Proficient bioactive ingredient extraction from plant samples is primarily reliant on the solvent type utilized in the extraction process. Most commonly, water is used as the extracting solvent by the traditional healer but a water and alcohol mixture is more effective for extracting most plant metabolites in the maceration technique of plant extraction [48]. The collected fresh *M. lanceolata* leaves were rinsed with pure water to get rid of impurities. The leaves were then dried for about 3 weeks under shade and crushed into a coarse powder using a mortar and pestle. The sample was weighed, and extraction was done by dispersing (soaking) 400 g of the plant material in 1600 ml of methanol (80%) for three days. Shaking at 120 rpm was carried out occasionally by a mechanical shaker. Next, the macerated plant material was filtered successively using gauze and Whatman filter paper-1 (Whatman, England). To obtain more yield, successive extraction of the residues was undertaken using 1600 ml of the same solvent. The filtrates were collected in one container, and methanol was removed by a rotary evaporator (R 200, Switzerland) under a temperature below 40°C and at 45 rpm. Then, the concentrated sample was further dried using a lyophilizer [49, 50]. Finally, the dried 80% methanolic extract was kept in a deep freezer (-20°C) until the experiment was carried out.

**Figure 2:**
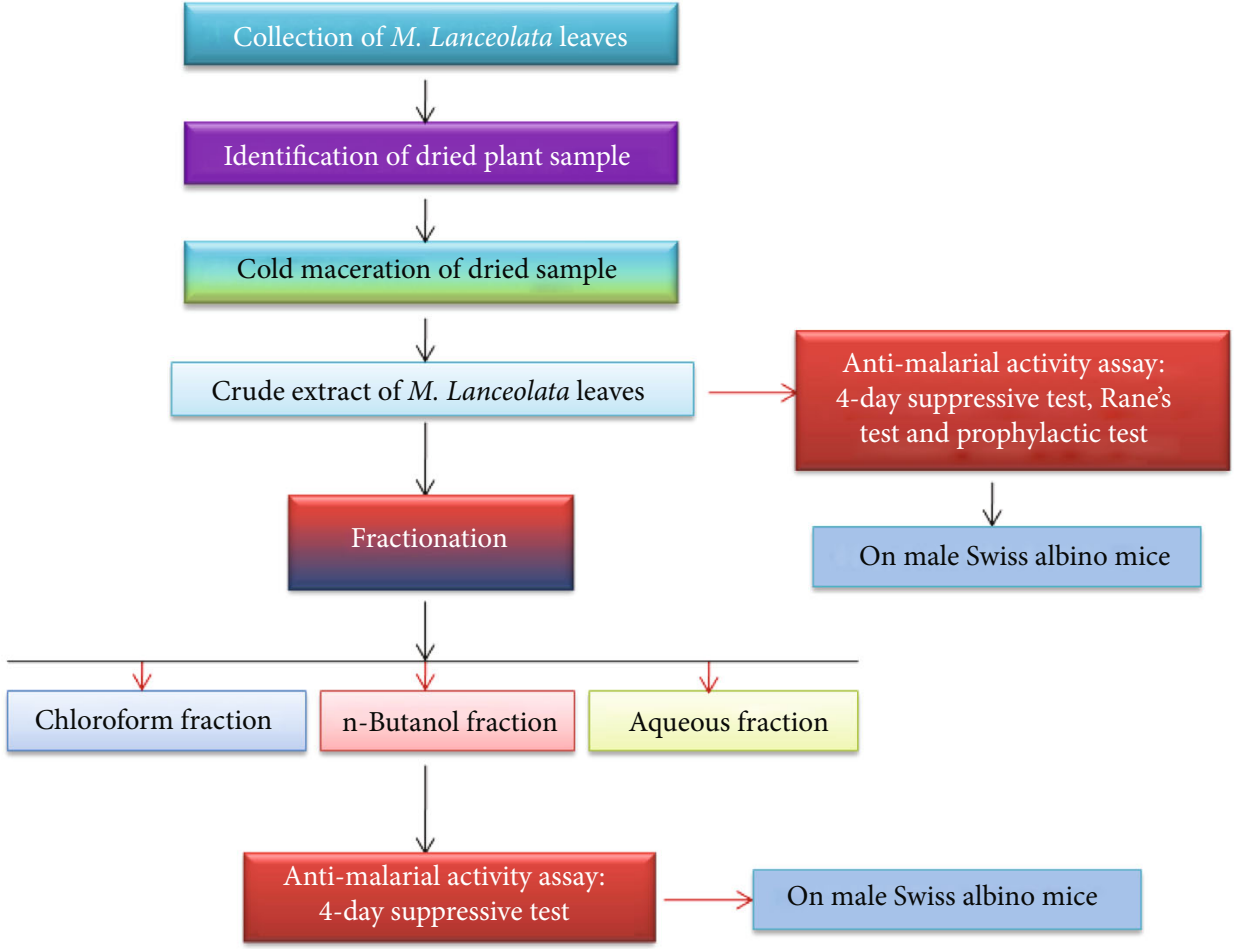
Schematic diagram of the experimental procedures.

The 80% methanolic extract was then partitioned using a fractionation method [51]. Solvent partitioning was conducted sequentially to obtain three different fractions. First, 44 g of the crude extract was suspended in 200 ml aqueous solvent using a separator funnel and shaken 3 times with 200 ml chloroform to get chloroform fraction. Then, the water layer was shaken 3 times with 200 ml n-butanol solution to obtain the n-butanol fraction. Finally, the remaining distilled water residue was taken as the last fraction. To get the concentrated extract, chloroform and n-butanol fractions were subjected to evaporation in rota vapor. The aqueous residue was frozen at -20°C for 24 h and then dried in a lyophilizer machine. The final concentrated filtrates (fractions) were kept at -2 to -8°C until used in the actual experiment (Figure 2).

### 2.4. Phytochemistry Analysis

To identify the secondary metabolites from methanol (80%) extract and solvent fractions, preliminary phytoconstituent screening tests were performed using officially approved procedures described in [52–54].

#### 2.4.1. Detection of Alkaloids

Each extract was dissolved separately in dilute hydrochloric acid (HCL). The dissolved substance was then filtered, and the filtrates were treated with Mayer's reagent (potassium mercuric iodide). The presence of alkaloids was evaluated through the formation of a yellow-cream precipitate.

#### 2.4.2. Detection of Anthraquinone

Each extract was hydrolyzed in diluted HCL and then treated with ferric chloride solution and immersed in boiling water for 5 minutes. The mixture was cooled and shaken with an equal volume of benzene. The separated benzene layer was then treated with an ammonia solution. The presence of anthranol glycosides was detected through the formation of rose-pink color in the ammonical layer.

#### 2.4.3. Detection of Flavonoids

Each extract was treated with little drops of lead acetate solution. The presence of flavonoids was asserted through the formation of a yellow color precipitate.

#### 2.4.4. Detection of Saponins

Each extract was diluted in 20 ml pure water and shaken in a graduated test tube for 15 min. The presence of saponins was assessed through the formation of a 1 cm layer of foam.

#### 2.4.5. Detection of Phytosteroids

Each extract was treated with chloroform solution. The dissolved material was then filtered, and the filtrate was treated with a few drops of acetic anhydride, boiled, and cooled. A few drops of concentrated sulphuric acid were added cautiously along the sides of the test tube. The presence of phytosterols was shown through the formation of a brown ring at the junction.

#### 2.4.6. Detection of Tannins

Gelatin 1% solution containing sodium chloride was added to each extract. The presence of tannins was indicated through the formation of a white precipitate.

#### 2.4.7. Detection of Terpenoids

Each extract was treated with chloroform solution. The dissolved extract was then filtered, and the filtrate was treated with a few drops of concentrated sulphuric acid. The mixture was shaken and allowed to stand. The presence of triterpenes was approved through the appearance of golden yellow color.

### 2.5. *In Vivo* Acute Oral Toxicity Assessment

For both 80% methanolic crude extract and its fractions, an acute oral toxicity test was done using standard protocols [55]. Twenty healthy female mice (nonpregnant) at age of 6 to 8 weeks were used in this procedure. Every mouse fasted for 4 h prior to and 2 h subsequent to oral administration of the test drugs. First, a limited test dose of the test drugs (2000 mg/kg) was provided by oral gavage for one mouse from each group. Each mouse was then followed for 24 h for gross changes in behavior (hair erection, convulsion, poor appetite, lacrimation, tremor, salivation, diarrhea, mortality) after administering the test substances. Since either death or gross behavioral changes were not seen in the first mouse, the same dose was given to another four mice. Each mouse was given a single dose and was observed for 4 h with 30 min intervals and then for 14 successive days once daily for clinical pictures of toxicity. The doses of the test drugs were then determined as 1/20^th^, 1/10^th^, and 1/5^th^ by considering the data obtained from acute toxicity tests and a pilot study [56]. Thus, 3 doses (200, 400, and 600 mg/kg) were chosen for all test substances.

### 2.6. Skeleton of the Study

The study animals were arbitrarily allocated into 5 groups, each consist of 6 mice housed in one cage. The first three categories (groups I, II, and III) were orally treated with 200, 400, and 600 mg/kg of either hydromethanolic leaf extract or solvent fractions, respectively, while the second categories (groups IV and V) were treated with the diluents for negative control (10 ml/kg pure water for crude extract, n-butanol fraction, and distilled water fraction but 10 ml/kg 2% Tween-80 for chloroform fraction) and standard drug (chloroquine 25 mg/kg) for positive control, respectively. The maximum volume of fluid given to the study mice was 10 ml/kg.

### 2.7. Inoculation of Malaria Parasite

For each test, the parasitized red blood cells were taken from *P. berghei*-infected donor mice with a parasitemia of 20 to 30%. The donor mice were surrendered by a head-blown procedure, and venous blood was taken in a Petri dish with 0.5% trisodium citrate (BDH chemicals, England) through the incision of a jugular vein. The venous blood was then thinned with normal saline (0.9%) in 1 to 4 proportions to get a final suspension that holds 1 × 10^7^ infected erythrocytes in every 0.2 ml. The dilution was made based on the parasitemia of donor mice and erythrocyte count of the normal mice in such a way that one ml of blood contains 5 × 10^7^ infected erythrocytes. Every experimental mouse was then infected through the intraperitoneal route with 0.2 ml suspension [48, 50].

### 2.8. Chemosuppressive Model

The four-day suppressive model described by David, Thurston, and Peters was used for assessing the schizonticidal effects of 80% methanolic crude extract and its fractions on early infection [57]. On day 1 (D0), 2 h before treatment, test mice were infected with 0.2 ml suspension of parasitized venous blood intraperitoneally. As described above in Section 2.6, the test crude drugs were provided orally to the test animals once daily (every 24 h) for 4 days, while the positive and negative control groups received standard and placebo drugs, respectively. Blood was then taken from 0.5 to 1 mm segment of the mice's tails on the 5^th^ day (D4) to make a blood film and measure parasite levels. On D0 before infection and at D4, packed cell volume (PCV), body weight, and rectal temperature were measured and documented.

### 2.9. Rane's Test (Curative Model)

As usual, 30 mice were divided by chance into 5 groups with 6 in each group to assess the curative effects of the hydroalcoholic leaf extract of *M. lanceolata* following the procedure outlined by Ryley and Peters [58]. On the first day (D0), the animals were infected with infectious venous blood. After 3 days (72 h postinoculation), the mice grouped were treated accordingly (see Section 2.6) every 24 h for four successive days. The tail blood was taken from every mouse, and thin blood films were prepared on day 4 (D3) before treatment and on day 8 (D7) to measure and calculate percent suppression and parasitemia levels. Measurement of PCV, body weight, and rectal temperature was carried out for every mouse prior to the provision of the first dose at D3 and after the completion of treatment at D7.

### 2.10. Residual Infection Model

The prophylactic potential of 80% ME was further evaluated according to the repository model written by Peters [59]. Weighed study mice were assigned arbitrarily into 5 groups and treated as illustrated in Section 2.6 for 4 successive days. At D4 (on day 5), study animals were infected with *P. berghei* and followed for 3 days. After 72 h of infection on day 8 (D7), parasitemia and suppression activity of the extract were determined. Data regarding the hematocrit, body weight, and body temperature of the experimental mice have gathered ahead of inoculation at D4 and D7 (end of the test).

### 2.11. Parasitemia Level Determination

A drop of peripheral blood was received by frosted microscopic slides from the tail cut of every mouse, and blood smears (thin) were made, fixed with 99% methanol, and stained with Giemsa stain (10%) at pH around 7.2 for fifteen min. The slides were then rinsed with pure water and exposed to environmental temperature for air drying. From each stained microscopic slide, 5 fields were erratically selected and observed under a microscope with an oil immersion through ×100 objective. The percent of parasitemia was decided by counting infected erythrocytes out of 200 red blood cells on arbitrarily chosen fields of the frosted microscopic slide. Parasitemia level (% parasitemia) and percentage of suppression were quantified using the formulas shown below [60].

(1) $$\begin{array}{c} \%\text{parasitemia}=\frac{\text{Number of infected erythrocytes}}{\text{Total number of erythrocytes counted}}\text{ x }100,\\ \%\text{of suppression}=\frac{\left(\text{Parasitemia in the negative control}-\text{parasitemia in treated group}\right)}{\text{Parasitemia in negative control}}\text{ x }100. \end{array}$$

### 2.12. Mean Survival Day Determination

The mortality of mice was checked every day, and days from parasite inoculation up to mortality were ticked for every mouse in all study groups during the follow-up time (28 days). The mean survival days for every group were calculated according to [50]:

(2) $$\begin{array}{c} \text{Mean survival days}=\frac{\text{Sum of survival days of mice in group}}{\text{Total numbers of mice in that group}}. \end{array}$$

### 2.13. Mean Body Weight and Temperature Determination

The rectal temperature and body weight were determined by recording the average temperature and weight of study animals in every experimental group and comparing them with the respective negative control. The body weight and temperature of every study mouse in every group were taken utilizing a sensitive digital weighing balance and thermometer (rectal), respectively. The changes in mean results reported in % before and after treatment were then calculated [48, 50].

### 2.14. Mean Hematocrit Determination

From each mouse, tail blood was taken by heparinized capillary tubes. Three-fourths of each tube was filled with tail blood and sealed by sealant clay. The sealed tube was then sited in a standard centrifuge with the closed tips outward and centrifuged for five min at 12,000 rpm. The total blood volume and volume of red blood cells were determined using a hematocrit reader, and hematocrit (PCV) was quantified using the formula illustrated below [50].

(3) $$\begin{array}{c} \text{Packed cell volume}=\frac{\text{Volume of erythrocyte in a given blood volume}}{\text{Total blood volume}}\text{x }100. \end{array}$$

### 2.15. Data Quality Control

All equipment, reagents, chemicals, and materials used were of analytical grade. Randomization was used during experimental animal grouping and assignments of treatments. Data quality was also controlled by a pilot study, acclimatizing the study mice, following protocols strictly, and coding all frosted slides (microscopic). Infected red blood cells were enumerated blindly by laboratory professionals. Additionally, exterior factors were decreased by the utilization of naive animals housed in a favorable laboratory environment. The animal keeper kept the sanitation of the cages every other day.

### 2.16. Data Interpretation

The obtained facts were cleared, structured, entered into Microsoft Excel 2010, and exported to SPSS version 22. The result was presented as the mean and standard error of the mean (SEM). ANOVA (one-way) with Tukey's post hoc test was employed to determine the statistical significance of the mean parasitemia suppression, survival days, body weight, rectal temperature, and packed cell volume between the groups. All data were evaluated with a confidence interval of 95%. The *P* value <0.05 was statistically significant.

## 3. Results

### 3.1. Extract Yields

At the end of crude extraction, a total yield of 71.9 g of 80% methanol extract was obtained. Forty-four grams of this extract was partitioned (fractionated) using chloroform, n-butanol, and water solutions. The percentage yields were depicted here (Table 1).

### 3.2. Phytochemistry Screening

The finding of the preliminary phytoconstituent analysis on the 80% methanolic extract showed the existence of most screened metabolites except alkaloids and terpenoids (Table 2). Tannins and flavonoids were present in every solvent fraction, whereas steroids were revealed in both chloroform and water fractions. Moreover, among fractions, saponins were present in both n-butanol and water fractions but alkaloids were seen in the chloroform fraction, anthraquinone in the water fraction, and terpenoids were detected in the n-butanol fraction.

### 3.3. *In Vivo* Assay of Acute Oral Toxicity

The conducted toxicity study revealed that the lethal dose (LD_50_) of the hydroalcoholic extract of all fractions was above 2000 mg/kg. All test samples that were given orally to study mice in a single dose of 2000 mg/kg caused no morbidity and mortality within the observation period. No visible signs of behavioral and physical changes were seen in the study mice.

### 3.4. Bioactivity of Methanolic (80%) Extract and Its Fractions in Four-Day Chemosuppressive Model

In the four-day test, the 80% methanol extract and its fractions exhibited a noteworthy (*P* < 0.001) reduction of parasite at various doses as compared to the respective vehicle used for reconstitution. The highest parasite reduction (81.28%) has been shown by chloroform fraction (CF) at 400 mg. However, the effects produced were significantly (*P* < 0.001) smaller than that of the standard chemical. Chloroquine (CQ) cleared the parasite to untraceable levels (Table 3).

Among the doses of CF, the 400 mg showed a significant difference (*P* < 0.001) in chemosuppression as compared to the 200 and 600 mg. In the same fashion, the n-butanol fraction (BF) and distilled water fraction (DWF) showed a significant difference (*P* < 0.001 and *P* < 0.05, respectively) in the inhibition of parasites when a comparison was made between the respective 200 and 600 mg. Moreover, BF demonstrated a remarkable (*P* < 0.05) parasite suppression at 400 mg as compared to 200 mg. The lowest parasite suppression (31.28%) was seen at 200 mg of DWF (Table 3).

In this early infection test, the analysis displayed a strongly significant (*P* < 0.05 to *P* < 0.001) improvement of the mean survival time (MST) in days with the 80% methanol and solvent fraction treatment. The 600 mg of the crude leaf extract (ML) (*P* < 0.05), all doses of CF (*P* < 0.001), all BF doses (*P* < 0.001 for 400 and 600 mg and *P* < 0.01 for 200 mg), and all doses of DWF (*P* < 0.001) were able to increase survival days considerably as compared to the respective placebo substance (negative control). Groups treated with CQ had significantly (*P* < 0.001) longer mean survival days compared to the respective pure water (PW) and extract-treated groups. In BF and DWF, the 600 mg outlasted the respective 200 mg but the 400 mg outlasted the respective 200 and 600 mg in CF, with significant (*P* < 0.001, *P* < 0.01, *P* < 0.001, and *P* < 0.01, respectively) survival days. Additionally, the 400 mg of DWF produced an appreciably (*P* < 0.01) increased survival date as compared to the respective 200 mg. The standard chemical exhibited a larger (*P* < 0.001) prolonged survival time (28 days) than methanol (80%) extract and solvent fractions (Table 3).

Animals treated with all 80% methanol extract doses were unable to protect against parasite-induced weight loss in the 4-day chemosuppressive test (Table 4). In contrast, CQ protected body weight reduction significantly (*P* < 0.001 and *P* < 0.01) as compared to the pure water and extract doses, respectively. From solvent fractions, only 400 mg CF significantly (*P* < 0.05) prevent weight loss compared to the placebo substance. The standard drug provided significant protection against a decline in body weight as compared to the 200 mg of CF and each dose of both BF and DWF with different levels of *P* value. The crude extract did not have any effect on the protection of rectal temperature drop in the four-day test. Coming to solvent fractions, the 400 mg of CF (*P* < 0.01), all BF (*P* < 0.001), and DWF doses (*P* < 0.001 for 600 mg and *P* < 0.01 for 200 and 400 mg) protected a considerable reduction of body temperature as compared to the respective placebo drugs. When a comparison was made between doses of CF, 400 mg averted a significant fall in body temperature related to 200 mg. Chloroquine exhibited promising activity in the control of rectal temperature lessening relative to the 200 mg of CF and negative control of CF, BF, and DWF treated groups with *P* < 0.01, *P* < 0.01, *P* < 0.001, and *P* < 0.001, respectively (Table 4).

Table 5 shows that attenuation of anemia in the four-day suppressive model was obtained by the 400 and 600 mg of CF as compared to 2% tween-80 with the level of significance of *P* < 0.01 and *P* < 0.05, respectively. A noteworthy difference (*P* < 0.05) has also been seen when the effect resulting in the prevention of PCV reduction by 600 mg of BF was compared to the placebo treatment. No detectable alterations were noted with any ML and DWF doses as compared to the pure water effect (Table 5).

### 3.5. Bioactivity of 80% Methanol Extract in Curative Model

**Figure 3:**
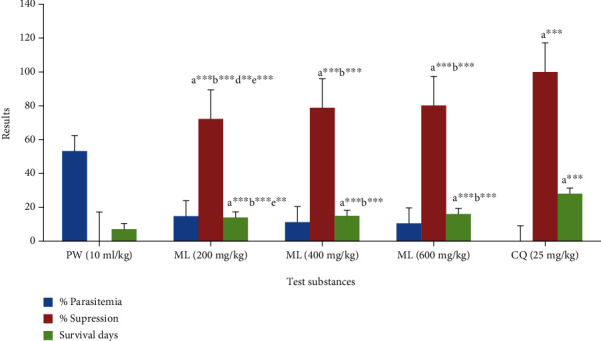
Bioactivities of 80% methanol extract of *M. lanceolata* leaves against rodent malaria parasite in the curative test. Values are designated as mean ± SEM (*n* = 6). a compared to PW, b to CQ, d to 400 mg of test substance, and e 600 mg of test substance: ^∗∗^*P* < 0.01, ^∗∗∗^*P* < 0.001; PW: pure water (negative control); ML: 80% methanol extract of *M. lanceolata*; CQ: chloroquine.

The crude extract was evaluated for its curative effects as shown in Figure 3. The mean parasitemia was larger in the vehicle-treated groups than in the extract-treated groups; meaning, each dose of the methanol (80%) extract drastically (*P* < 0.001) suppressed percentage parasitemia as compared to the vehicle material. A decrease in parasite level by chloroquine was statistically significant (*P* < 0.001) when compared to a negative control agent and every dose of the tested drugs. Additionally, statistically noteworthy discrepancies (*P* < 0.01 and *P* < 0.001) were seen in suppressing parasites when the 200 mg of ML was compared to the 400 and 600 mg of ML. The survival period was altered significantly (*P* < 0.001) by CQ and all extract doses relative to PW in Rane's test. Statistically considerable (*P* < 0.001) prolonged survival days were also found concerning comparison among CQ and doses of the extract. Nevertheless, the increase attained with ML was not high as that of CQ.

The crude extract demonstrated a deterrent effect on weight and temperature drop in infected animals in the established infection test (Table 6). The weight of the mice group that took CQ and 600 mg of ML were found to be notably (*P* < 0.001 and *P* < 0.01) improved as compared to the PW. Among extract doses, ML 600 mg had a meaningful outcome (*P* < 0.05) in weight improvement relative to ML 200 mg. Chloroquine was also drastically (*P* < 0.01 and *P* < 0.001) protected weight loss as compared to both 400 and 200 mg of ML. Chloroquine (*P* < 0.001) and both ML 600 mg (*P* < 0.001) and ML 400 mg (*P* < 0.05) showed statistically imperative protection from a reduction in body temperature as compared to the placebo principle. The highest encouraging result on the protection of rectal temperature falls with a significant value (*P* < 0.001) was reported in mice treated with the standard chemical (positive control) during the curative test as compared to 200 and 400 mg of ML. The 600 mg of methanol 80% extract averted temperature loss appreciably (*P* < 0.001) relative to its 200 mg dose (Table 6).

Analysis of PCV among the groups receiving extracts and controls in the curative model revealed that CQ (*P* < 0.001) and both 600 (*P* < 0.001) and 400 mg (*P* < 0.05) of ML were capable to prevent fall in PCV appreciably as compared to the solvent (a constituent), with the highest effect by CQ. A significant difference (*P* < 0.05) was also attained in averting falls in PCV by standard substance relative to the 200 mg of ML (Table 7).

### 3.6. Bioactivity of 80% Methanol Extract in Chemoprophylactic Test

**Figure 4:**
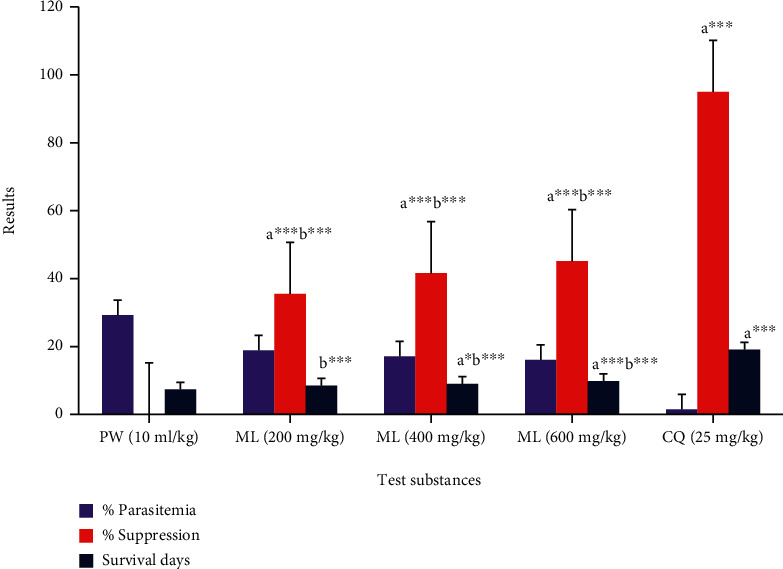
Bioactivities of 80% methanol extract of *M. lanceolata* leaves against rodent malaria parasite in the prophylactic test. Values are designated as mean ± SEM (*n* = 6). a compared to PW, b to CQ,: ^∗^*P* < 0.05, ^∗∗∗^*P* < 0.001; PW: pure water (negative control); ML: 80% methanol extract of *M. lanceolata*; CQ: chloroquine.

CQ and every ML dose suppressed parasite load significantly (*P* < 0.001) relative to the PW in the prophylactic test (Figure 4). Statistically significant (*P* < 0.001) suppression of the parasite was also produced by the standard chemical as compared to each extract dose, though absolute eradication was not attained. The survival period of the mice (infected) pretreated with the methanol (80%) leaf extract in the chemoprophylactic test indicated that both the 600 and 400 mg of ML were accomplished significantly (*P* < 0.001 and *P* < 0.05, respectively) prolonged survival days as compared to the placebo control. Maximum prolongation (*P* < 0.001) of MST has also been shown by chloroquine relative to the vehicle and all extract doses (Figure 4).

The standard drug and 600 mg of methanol (80%) extract showed a significant (*P* < 0.01 and *P* < 0.05, respectively) protective effect in weight (body) reduction as compared to pure water in the residual infection test (Table 8). The standard agent and both the 600 and 400 mg of ML were appreciably (*P* < 0.01, *P* < 0.01, and *P* < 0.05, respectively) prevented body temperature reduction relative to the placebo control. The 200 and 400 mg of ML exhibited significantly (*P* < 0.01 and *P* < 0.05) less effect than CQ in attenuating rectal temperature decline.

A comparison of PCV results (Table 9) in the repository model indicated that the standard control was able to attenuate PCV reduction in a statistically considerable (*P* < 0.01) way relative to the PW. CQ 25 mg also displayed a significant (*P* < 0.05) protection against anemia as compared to 200 mg of ML.

## 4. Discussion

Malaria is still the major parasitic disease in the world responsible for the mortality of more individuals than most other infectious or transmissible diseases [61]. Treating large numbers of malaria-infected people has resulted in extensive resistance to the present antiplasmodial bullets. Hence, there is an urgent need for innovation and the development of novel antiplasmodial drugs to resolve this challenge [62]. Natural antimalarial products obtained from medicinal plants are considered a leading strategy to solve this problem [63]. Therefore, evaluating the antimalarial activity of plant constituents is very important to contribute to the finding of lead chemical(s) [64].

The hydroalcoholic solvent was employed for the maceration of the investigational plant material because methanol (80% methanol) is a universal solvent and can extract most components (polar and nonpolar) of the plant samples [65]. Methanol has high efficiency in penetrating the plant cell wall, and a hydroalcoholic mixture could partition various compounds (with different polarity) than absolute methanol [66]. This could also be asserted based on the earlier scholarly papers that hydromethanol could be a superior solubilizing solution for extraction for *M. lanceolata* [18, 26, 34, 39].

In the current study, alkaloids, saponins, anthraquinones, flavonoids, tannins, steroids, and terpenoids were noticed in the leaf of the study plant during phytochemical analysis. The presence of those metabolites including phenols in different parts of *M. lanceolata* was also reported by other researchers in previous works [18, 34]. There were no mice that showed signs of toxicity within 24 h and the next 14 days posttreatment with 2000 mg/kg of the test substances during *in vivo* acute oral toxicity assay. A greater than 2000 mg/kg oral dose is ten times greater than the lowest effective dose of the test chemicals. Previous studies have shown that a test substance is a strong candidate for further investigation if the lethal dose 50 (LD_50_) of the substance is three times higher than the lowest effective dose [67, 68]. Previous studies also reported that extracts of *M. lanceolata* were relatively nontoxic [33–36]. Moreover, the methanolic leaf extract of *Lophira lanceolata* (with the same species as the study plant) is safe up to a 5,000 mg/kg dose [69]. These findings could enlighten the safe use of *M. lanceolata* to manage malaria by Ethiopian citizens.

Nonprimate animals are not invaded by species of malaria parasites that bring disease to humans. As a consequence, rodent parasites such as *P. berghei* are used to test antiplasmodial compounds. The rodent model of malaria has been effectively validated, and an *in vivo* study was selected because prodrug effects and the possibilities of infection control by the immune system are taken into consideration. The four-day chemosuppressive model is the primary and most commonly used test for the evaluation of innovative antiplasmodial drugs [48, 70].

In this experiment, it was approved that methanolic (80%) leaf extract of *M. lanceolata* and its fractions possess antiplasmodial activity at all doses in the 4-day chemosuppressive study. This might be secondary to the suppressive result of the test substances on the multiplication and erythrocyte infectivity of the parasite. Yet, the outcome was lower than the standard substances. This probably is due to the crude nature of the plant material. The demonstrated *in vivo* bioactivity of the study plant is supported by its previous *in vitro* antiplasmodial activities [38, 39]. This avowal is additionally supported by the antiplasmodial (*in vivo*) effects of other genera of the same species *Lophira lanceolata* [69]. Although they have yet to be examined, the benzoquinone components of *M. lanceolata* [13, 25] might be responsible for its activity since benzoquinones of the Myrsinaceae family are known for their antimalarial effects [71]. Antibacterial drugs like clindamycin and doxycycline are utilized in the chemotherapy of plasmodium species. Hence, the *in vitro* antibacterial activities of *M. lanceolata* confirmed previously [18, 34–36] further support the current result.

In the four-day test, parasitemia load reduction was increased as the 80% methanol (crude) extract dose increased. The highest inhibition was 51.31% at a 600 mg dose. This finding is in line with the parasitemia suppression of crude extract of *Hypoestes forskalei* (51.28%) at 400 mg dose [72] and *Myrica salicifolia* (51%) at 200 mg [56]. Although the test doses are different, the leaf extract of *Calpurnia aurea* displayed comparable parasite suppression with our study plant [73]. Moreover, the 200 mg of *M. lanceolata* hydroalcoholic extract exhibited almost similar parasite suppression (42.24%) to that of the 735 mg of 70% ethanolic extract of *Carpolobia lutea* (42.54%) [74].

All solvent fractions of *M. lanceolata* significantly reduced the parasitic load in infected mice during the 4-day test. Among them, the chloroform fraction exerted the highest suppressive activity (81.28%) at 400 mg. A study conducted in Uganda reported a similar finding in which chloroform extract had the highest *in vitro* antiplasmodial activity [38]. The effect revealed by the chloroform fraction overwhelms the effect exhibited by the crude extract. This is probably because the activities of one chemical found in the crude extract may mask the other. Thus, fractionation may aid to eliminate toxic, inactivating compounds [75, 76]. The 400 mg of *M. lanceolata* chloroform fraction demonstrated almost the same amount of parasite suppression (81.28%) as that of the 14 mg of *C. lutea* chloroform fraction (81.35%) [74]. The current finding is in agreement with *Maytenus gracilipes*, *Croton macrostachyus*, and *Vernonia amygdalina* in which the chloroform fraction produced a more suppressive effect than the remaining fractions [48, 49, 77].

The peak activity produced by chloroform fraction could tell us that the nonpolar ingredients are more effective than the medium-polar and highly polar constituents. Most probably, this activity could be attributed to myrcene isolated from chloroform extract of Ethiopian *M. lanceolata* leaves [24]. In contrast, the n-butanol fraction showed better suppression than chloroform and water fractions as shown in other studies done on other plants [50, 72, 78–80]. Such variable findings might be due to the difference in the concentration level of the bioactive ingredients in the crude extract and solvent fractions [81]. The 600 mg of chloroform fraction has lower efficacy than the 400 mg. This might probably be due to the ingredients in the fraction that may pursue dissimilar kinetics [66]. The percentage suppression of parasitemia showed by 400 (41.84%) and 600 mg (46.11%) of the n-butanol fraction is in line with the result exhibited by 200 (41.06%) and 400 mg (45.52%) of the same fraction of *H. forskalei* [72].

The survival day of the mice infected with the *Plasmodium* parasite is among the assessed parameters to evaluate the antiplasmodial activity of claimed medicinal plants [56]. Plant extract that caused survival days of treated infected mice greater than nontreated infected mice was considered active [82]. In a 4-day test, the longer survival day was displayed by each dose of all solvent fractions and only the 600 mg of the 80% methanol leaf extract relative to the placebo control. This finding indicates that the test product had active agents, and the effect was strongly associated with the suppressive action of the plant. The 400 mg of chloroform fraction resulted in a maximum survival period (18 days) than other doses of the tested drug. This is consistent with the parasitemia suppression effect of this chloroform dose. The mean survival time (15 days) resulting from the 400 mg of chloroform fraction is similar to the result created by the 600 mg of *H. forskalei* crude leaf extract [72]. Besides being active agents, the plant ingredients might play the role of different survival times among the treated mice as survival days depend on the concentration and other properties of plant ingredients [83]. The survival days of the extract and fraction-treated mice were shorter than the survival days of mice treated with chloroquine. This might possibly be due to the quick reversible action (due to rapid metabolism) or the rapid elimination phase of the test substances [84].

All methanolic (80%) leaf extract doses did not avert body weight loss in the suppressive study. This finding is in line with the effect shown by *Myrica salicifolia* [56] but is contrary to the result produced by *H. forskalei* [72]. From fractions, only the 400 mg of chloroform fraction reversed weight (body) reduction possibly due to a significant reduction of parasitemia level. Before the death of mice, there is a decline in metabolic rate followed by a fall in body temperature dissimilar to the conditions happening in humans [67]. Active substances should protect against the quick dropping of body temperature. However, antipyretic drugs (a plant extract with an antipyretic constituent) may induce or exacerbate hypothermia in humans and mice [85, 86]. Contrary to [72], the hydromethanolic extract was not averted temperature drop in the four-day test. This might be contributed to the pronounced hypothermic effects of the test substance. Of course, the hydroethanolic *M. lanceolata* leaf extract significantly reduced rectal temperature in rats [37]. In contrast to this, the 600 mg of chloroform fraction and all doses of n-butanol (in line with [77]) and water fraction protected a fall in body temperature (in line with [72]). This protective activity might be due to a low concentration of the hypothermic constituents in the fractions or amelioration of some pathogenic conditions [87]. The 600 and 200 mg of chloroform fraction and the 600 mg of n-butanol fraction reversed the occurrence of anemia in malaria-infected mice in early infection tests. This might be related to (1) the existence of metabolites in those fractions to stabilize erythrocytes and (2) the significant reduction of parasitemia levels because the increase in blood parameters is frequently correlated with the reduction in parasitemia burden [88]. However, the methanol 80% leaf extract did not avert the reduction in packed cell volume, which is in line with the 80% methanolic leaf extract of *M. salicifolia* [56].

The crude hydromethanolic extract of *M. lanceolata* decreased parasitemia levels on established infections in the curative model. The outcome of the testing effect in mice given 200, 400, and 600 mg demonstrated that the suppressive activities (72.26%, 78.80%, and 80.22%, respectively) were in a dose-dependent mode like root barks of *Gardenia ternifolia* [78] and leaves of *M. gracilipes* [48]. As this extract has bioactivity in both chemosuppressive and curative methods, we can take this experimental plant as a future source in developing novel antiplasmodial compounds [89].

The parasite suppressive activity that occurred in the established infection test was higher than the activity that occurred in the early infection test. This is in concurrence with reports on the stem bark of *Faidherbia albida* [90] and the root bark of *Alstonia boonei* [91]. Such types of effects might be ascribed to the suppressive action of the crude extract on the proliferative phase of *P. berghei*. The access of *Plasmodium* species to the erythrocytes alone does not generate a disease. Generation of free radicals, activation of phospholipase cascade, and production of prostaglandins by the immune (host) system in response to foreign disease-causing germs are also involved in producing disease [90]. Accordingly, the outstanding curative activity (observed in Rane's test) than the schizonticidal activity might be due to the inhibition action of the test substance on the generation of hemolytic and free radical molecules by the malaria parasite [92]. The antioxidant activity of this medicinal plant, *M. lanceolata*, has been scientifically proven in another experiment [36]. It acts by removing reactive oxygen species by increasing the amount of superoxide dismutase, glutathione, and catalase enzymes [37]. This action is most likely contributed by the availability of phenols and tannins (protective of the body's endogenous antioxidant enzymes) in the study plant [34, 92]. Phenolic metabolites are famous for their antioxidant properties through trapping reactive oxygen and radical species as well as the neutralization of free radicals [93]. Moreover, the isomeric acylated benzoquinones isolated from *M. lanceolata* have produced remarkable antioxidant and antiproliferative activity [25].

The crude extract substantially prolonged the mouse survival in the curative test. This justifies that parasite reduction is closely correlated with the longer survival of the mice. Our study plant has anti-inflammatory activities [16], which might be ascribed to the antimalarial effects. This activity could potentially be produced by an inhibitory activity of maesanin (a compound isolated from *M. lanceolata*) on the 5-lipoxygenase enzyme [32]. As indicated in a recent investigation, the additional possible mechanism of action by the active compounds of the study plant could be through increasing the level of circulating white blood cells [37]. The phytosteroids, saponins, and flavonoids identified in this experimental plant are known for their immune system modulation [94, 95]. The survival days resulting from the 200 and 400 mg of the study plant were in agreement with the mean survival time exhibited by 490 and 735 mg of *C. lutea* during Rane's test [74].

In contrast to the result seen in the chemosuppressive study, the extract of *M. lanceolata* averted loss in weight (only at 600 mg) and a drop in mice temperature (at 400 and 600 mg) in the curative test. This outcome is in line with the 80% methanolic extract of *M. salicifolia* leaves [56]. This could be brought through the superior suppression of parasites and more prolongation of mice survival in Rane's test. The attenuation of decline in packed cell volume by the crude extract in established infection could be due to the destructive antimalarial activity against invaded erythrocytes and the malaria parasite, thereby sustaining the accessibility of new erythrocytes. It was proved in another study that the study plant extract can reduce anemia by increasing the level of erythrocytes [37]. Its antioxidant property could also safeguard red blood cells due to the inhibition of hemolytic factor generation [96].

In this study, the potential preventive effect of the study plant against parasitemia proliferation was tested by using the prophylactic study. The result of this study indicates that the plant extract showed prophylactic activity. The percent suppression of parasitemia by 80% methanolic *M. lanceolata* leaf extract at 200 mg (35.51%) is similar to the effect produced by 137 mg (35.47%) of crude root extract of *Panicum maximum* [97]. If the tested drugs suppress percent parasitemia by more than 30%, we can come up with a conclusion that the claimed medicinal plant has an antimalarial effect [70]. Even though the test substance significantly suppressed the parasite level, they were smaller than seen in the 4-day chemosuppressive and Rane's models. The same results, in which the test substances had better chemosuppressive and curative activity than prophylactic results, were reported in other research works [73, 78, 91]. This is probably due to the extract administration before the occurrence of the infection, the method used (*in vivo*), the way we inoculate the parasite, and the doses given that result in quick red blood cells invasion without the *Plasmodium* species passing the hepatic phases [98, 99]. Except at 200 mg, the crude extract drastically increased the survival days in the chemoprotective test relative to the pure water. This further proves the evidence that the test substances suppressed the parasite *P. berghei* ensuing in reduced pathogenicity [100].

The weight loss protective effect elicited by the 400 mg chloroform fraction in the suppressive model and by the 600 mg methanolic leaf extract in both curative and prophylactic models might partly be provided by the affirmed nutritional value of our plant [40]. In the residual infection, the 400 and 600 mg *M. lanceolata* leaf extract exhibited a protective effect against temperature reduction, which is in line with the effect of *M. salicifolia* roots [56]. Overall, the study plant byproducts alkaloids (intercalate with DNA and block cell division) [101], flavonoids (make a complex with intracellular soluble proteins), tannins (form complex with cellular proteins), terpenoids (cause membrane disruption) [18], saponins (form complex with cholesterol, glycolipids, and glycoprotein's in biological membranes) [66], and steroids (block the influx of important nutrients) [72] are well known by their antiplasmodial activity [89, 102, 103]. An antimalarial (*in vivo*) activity is categorized as moderate, good, and very good if the test substance exhibited % parasitemia suppression ≥ 50% at 500, 250, and 100 mg/kg dose per day, respectively [73]. Based on this categorization, a 200 mg/kg of methanolic leaf extract and chloroform fraction has provided drastic parasite suppression (>50%). It can thus be generally concluded that the study plant exhibited good antiplasmodial activity.

## 5. Conclusion

The study showed that *M. lanceolata* leaves possessed antimalarial activity. Chloroform fraction relatively showed the highest antiplasmodial activities, signifying that the nonpolar ingredients are responsible for its antiplasmodial (*in vivo*) effects. The outcome of this work offers the fact that it supports the earlier *in vitro* studies on the plant leaves as well as the claim mentioned by the local healers.

## Figures and Tables

**Table 1:** Yields of *Maesa lanceolata* leaf extract and its fractions.

Extract type	Actual yield	Percentage yield
Hydroalcoholic extract	71.9 g	17.98%
Chloroform fraction	14.26 g	32.41%
n-Butanol fraction	13.07 g	29.7%
Water fraction	16.67 g	37.89%

**Table 2:** Results of phytochemistry screening of *M. lanceolata* methanolic (80%) leaf extract and its fractions.

Phytosubstances	Dried leaves of *Maesa lanceolata*			
	ML	CF	BF	DWF
Terpenoids	−	−	+	−
Tannins	+	+	+	+
Steroids	+	+	−	+
Saponins	+	−	+	+
Flavonoids	+	+	+	+
Anthraquinone	+	−	−	+
Alkaloids	−	+	−	−

ML: methanol (80%) extract; CF: chloroform fraction; BF: n-butanol fraction; DWF: distill water fraction; +: present; -: absent.

**Table 3:** Bioactivities of *M. lanceolata* crude leaf extract and its fractions against the rodent malaria parasite in the four-day suppressive study.

Test substances	Dose/day	% parasitemia	% suppression	Mean survival days
PW	10 ml/kg	38.43 ± 1.18	0.00	7.42 ± 0.15

ML	200 mg/kg	22.19 ± 0.60	42.24^a^^∗∗∗^^b^^∗∗∗^	8.67 ± 0.80^b^^∗∗∗^
	400 mg/kg	20.36 ± 0.68	47.00^a^^∗∗∗^^b^^∗∗∗^	9.25 ± 0.44^b^^∗∗∗^
	600 mg/kg	18.71 ± 1.75	51.31^a^^∗∗∗^^b^^∗∗∗^	9.83 ± 0.48^a^^∗^^b^^∗∗∗^

CQ	25 mg/kg	0.00 ± 0.00	100.00^a^^∗∗∗^	28.00 ± 0.00^a^^∗∗∗^

2% TW-80	10 ml/kg	47.03 ± 2.85	0.00	6.83 ± 0.28

CF	200 mg/kg	16.77 ± 1.50	64.34^a^^∗∗∗^^b^^∗∗∗^^d^^∗∗∗^	14.00 ± 0.93^a^^∗∗∗^^b^^∗∗∗^^d^^∗∗∗^
	400 mg/kg	8.81 ± 0.54	81.28^a^^∗∗∗^^b^^∗∗∗^^e^^∗∗∗^	18.25 ± 0.38^a^^∗∗∗^^b^^∗∗∗^^e^^∗∗^
	600 mg/kg	15.55 ± 0.53	66.94^a^^∗∗∗^^b^^∗∗∗^	15.00 ± 0.37^a^^∗∗∗^^b^^∗∗∗^

CQ	25 mg/kg	0.00 ± 0.00	100.00^a^^∗∗∗^	28.00 ± 0.00^a^^∗∗∗^

PW	10 ml/kg	45.40 ± 2.50	0.00	7.25 ± 0.11

BF	200 mg/kg	29.58 ± 0.65	34.85^a^^∗∗∗^^b^^∗∗∗^^d^^∗^^e^^∗∗∗^	8.92 ± 0.40^a^^∗∗^^b^^∗∗∗^^e^^∗∗∗^
	400 mg/kg	26.41 ± 1.19	41.84^a^^∗∗∗^^b^^∗∗∗^	9.83 ± 0.36^a^^∗∗∗^^b^^∗∗∗^
	600 mg/kg	24.47 ± 0.48	46.11^a^^∗∗∗^^b^^∗∗∗^	10.92 ± 0.30^a^^∗∗∗^^b^^∗∗∗^

CQ	25 mg/kg	0.00 ± 0.00	100.00^a^^∗∗∗^	28.00 ± 0.00^a^^∗∗∗^

PW	10 ml/kg	46.18 ± 2.91	0.00	7.17 ± 0.11

DWF	200 mg/kg	31.74 ± 1.09	31.28^a^^∗∗∗^^b^^∗∗∗^^e^^∗^	8.83 ± 0.25^a^^∗∗∗^^b^^∗∗∗^^d^^∗∗^^e^^∗∗^
	400 mg/kg	27.71 ± 1.31	40.00^a^^∗∗∗^^b^^∗∗∗^	10.00 ± 0.34^a^^∗∗∗^^b^^∗∗∗^
	600 mg/kg	27.21 ± 1.34	41.09^a^^∗∗∗^^b^^∗∗∗^	10.08 ± 0.24^a^^∗∗∗^^b^^∗∗∗^

CQ	25 mg/kg	0.00 ± 0.00	100.00^a^^∗∗∗^	28.00 ± 0.00^a^^∗∗∗^

Values are designated as mean ± SEM(*n* = 6); ^a^ compared to PW, ^b^ to CQ, ^d^ to 400 mg/kg of test substance, and ^e^600 mg/kg of test substance: ^∗^*P* < 0.05, ^∗∗^*P* < 0.01, ^∗∗∗^*P* < 0.001; PW: pure water; ML: methanol (80%) extract; 2% TW-80: 2% tween 80; CF: chloroform fraction; BF: n-butanol fraction; DWF: distilled water fraction; CQ: chloroquine.

**Table 4:** Body weight and temperature of malaria-infected study mice treated with *M. lanceolata* methanol (80%) leaf extract and solvent fractions in the four-day chemosuppressive study.

Test substances	Dose/day	Body weight (g)			Rectal temperature (°C)		
		D0	D4	% change	D0	D4	% change
PW	10 ml/kg	31.65 ± 1.26	30.18 ± 1.31	−4.70	37.30 ± 0.24	35.40 ± 0.53	−5.10

ML	200 mg/kg	31.25 ± 1.79	30.35 ± 1.74	−2.85^b^^∗∗^	36.85 ± 0.42	35.50 ± 0.67	−3.52
	400 mg/kg	30.35 ± 1.29	29.70 ± 1.17	−2.07^b^^∗∗^	37.25 ± 0.16	36.00 ± 0.74	−3.35
	600 mg/kg	29.05 ± 1.69	28.65 ± 1.76	−1.40^b^^∗∗^	37.45 ± 0.38	36.50 ± 0.62	−2.54

CQ	25 mg/kg	30.00 ± 0.62	31.35 ± 0.72	4.49^a^^∗∗∗^	37.65 ± 0.13	37.90 ± 0.23	0.67

2% TW-80	10 ml/kg	25.60 ± 1.21	23.65 ± 1.23	−7.56	37.95 ± .13	36.65 ± 0.22	−3.42

CF	200 mg/kg	26.40 ± 0.76	25.40 ± 1.17	−4.02^b^^∗^	37.90 ± 0.19	36.72 ± 0.31	−3.13^b^^∗∗^^d^^∗^
	400 mg/kg	26.05 ± 0.75	25.95 ± 0.71	−0.35^a^^∗^	38.05 ± 0.20	37.88 ± 0.25	−0.44^a^^∗∗^
	600 mg/kg	25.50 ± 1.22	25.37 ± 1.44	−0.72	37.80 ± 0.29	37.03 ± 0.28	−2.02

CQ	25 mg/kg	28.10 ± 0.64	29.33 ± 0.72	4.37^a^^∗∗∗^	37.68 ± 0.18	37.70 ± 0.16	0.05^a^^∗∗^

PW	10 ml/kg	27.50 ± 1.06	25.40 ± 1.14	−6.27	37.00 ± 0.23	35.20 ± 0.26	−4.86

BF	200 mg/kg	28.50 ± 0.54	27.20 ± 0.54	−4.57^b^^∗∗∗^	37.50 ± 0.39	36.90 ± 0.39	−1.59^a^^∗∗∗^
	400 mg/kg	26.45 ± 1.11	25.40 ± 1.04	−3.76^b^^∗∗∗^	37.80 ± 0.15	37.30 ± 0.26	−1.33^a^^∗∗∗^
	600 mg/kg	27.05 ± 0.43	26.23 ± 0.49	−3.03^b^^∗∗^	37.70 ± 0.27	37.35 ± 0.27	−0.93^a^^∗∗∗^

CQ	25 mg/kg	28.55 ± 0.38	29.85 ± 0.63	4.52^a^^∗∗∗^	37.50 ± 0.24	37.53 ± 0.16	0.10^a^^∗∗∗^

PW	10 ml/kg	29.55 ± 0.74	27.29 ± 1.00	−6.94	36.88 ± 0.25	34.65 ± 0.34	−6.03

DWF	200 mg/kg	30.37 ± 0.91	28.63 ± 0.87	−5.65^b^^∗∗^	36.65 ± 0.25	36.20 ± 0.37	−1.23^a^^∗∗^
	400 mg/kg	31.97 ± 1.20	30.70 ± 0.81	−3.74^b^^∗^	37.58 ± 0.25	37.22 ± 0.32	−0.96^a^^∗∗^
	600 mg/kg	29.30 ± 0.27	28.30 ± 0.74	−3.43^b^^∗^	36.85 ± 0.17	36.52 ± 0.15	−0.89^a^^∗∗∗^

CQ	25 mg/kg	30.95 ± 0.45	32.48 ± 0.58	4.93^a^^∗∗^	37.57 ± 0.22	37.85 ± 0.19	0.76^a^^∗∗∗^

Values are designated as mean ± SEM (*n* = 6); ^a^ compared to PW, ^b^ to CQ 25 mg, ^d^ to 400 mg of test substance, and : ^∗^*P* < 0.05, ^∗∗^*P* < 0.01, ^∗∗∗^*P* < 0.001; PW: pure water 10 ml/kg (negative control); ML: crude extract of *M. lanceolata*; 2% TW-80: 2% tween 80; CF: chloroform fraction; BF: n-butanol fraction; DWF: distilled water fraction; CQ: chloroquine; D0: values on day 1 (pretreatment); D4: values on day 5 (posttreatment).

**Table 5:** Packed cell volume of malaria-infected study mice treated with *M. lanceolata* methanol (80%) leaf extract in the four-day chemosuppressive study.

Test substances	Dose/day	Packed cell volume		
		D0	D4	% change
PW	10 ml/kg	60.78 ± 2.04	41.80 ± 2.38	−30.68

ML	200 mg/kg	55.83 ± 2.73	40.73 ± 2.17	−25.82^b^^∗∗^
	400 mg/kg	61.45 ± 2.65	49.30 ± 2.33	−18.98^b^^∗^
	600 mg/kg	57.15 ± 2.94	50.65 ± 2.48	−10.56

CQ	25 mg/kg	58.42 ± 1.39	60.10 ± 1.09	2.97^a^^∗∗∗^

2% TW-80	10 ml/kg	46.50 ± 0.85	34.00 ± 1.44	−26.99

CF	200 mg/kg	50.83 ± 0.87	39.50 ± 1.52	−22.23^b^^∗∗∗^
	400 mg/kg	50.67 ± 1.36	44.33 ± 1.31	−12.40^a^^∗∗^^b^^∗∗^
	600 mg/kg	48.27 ± 1.76	40.25 ± 1.18	−16.21^a^^∗^^b^^∗∗∗^

CQ	25 mg/kg	59.64 ± 1.84	61.70 ± 1.79	3.51^a^^∗∗∗^

PW	10 ml/kg	53.12 ± 1.27	46.25 ± 1.41	−12.85

BF	200 mg/kg	51.82 ± 1.09	47.80 ± 1.40	−7.75^b^^∗∗^
	400 mg/kg	51.00 ± 0.97	47.60 ± 1.19	−6.66^b^^∗∗^
	600 mg/kg	52.00 ± 1.06	49.15 ± 0.96	−5.45^a^^∗^^b^^∗∗^

CQ	25 mg/kg	59.73 ± 0.99	61.96 ± 1.25	3.74^a^^∗∗∗^

PW	10 ml/kg	55.85 ± 1.37	49.75 ± 1.18	−10.58

DWF	200 mg/kg	57.94 ± 1.15	53.07 ± 1.25	−8.39^b^^∗^
	400 mg/kg	51.18 ± 1.28	47.19 ± 1.28	−7.71^b^^∗^
	600 mg/kg	53.52 ± 0.84	49.94 ± 1.22	−6.63^b^^∗^

CQ	25 mg/kg	60.82 ± 0.82	62.28 ± 0.79	2.41^a^^∗∗^

Values are designated as mean ± SEM (*n* = 6); ^a^ compared to PW, ^b^ to CQ 25 mg,: ^∗^*P* < 0.05, ^∗∗^*P* < 0.01, ^∗∗∗^*P* < 0.001; PW: pure water (negative control); ML: crude extract of *M. lanceolata*; 2% TW-80: 2% tween 80; CF: chloroform fraction; BF: n-butanol fraction; DWF: distilled water fraction; CQ: chloroquine; D0: values (pretreatment) on day 1; D4: values (posttreatment) on day 5.

**Table 6:** Body weight and temperature of malaria-infected study animals treated with *M. lanceolata* 80% methanol leaf extract in Rane's study.

Test substances	Dose/day	Body weight (g)			Rectal temperature (°C)		
		D3	D7	% change	D3	D7	% change

PW	10 ml/kg	29.75 ± 1.85	25.45 ± 1.41	−14.27	37.25 ± 0.44	32.37 ± 0.09	−13.05

ML	200 mg/kg	31.90 ± 1.90	27.70 ± 0.96	−12.49^b^^∗∗∗^^e^^∗^	37.50 ± 0.18	33.20 ± 0.29	−11.46^b^^∗∗∗^^e^^∗∗∗^
	400 mg/kg	31.65 ± 1.72	28.43 ± 1.30	−9.91^b^^∗∗^	37.85 ± 0.47	34.80 ± 0.22	−8.00^a^^∗^^b^^∗∗∗^
	600 mg/kg	30.20 ± 1.46	28.53 ± 1.19	−5.36^a^^∗∗^	37.52 ± 0.49	35.95 ± 0.23	−4.11^a^^∗∗∗^

CQ	25 mg/kg	30.27 ± 1.15	29.95 ± 1.13	−1.04^a^^∗∗∗^	37.83 ± 0.25	37.58 ± 0.14	−0.64^a^^∗∗∗^

Values are designated as mean ± SEM (*n* = 6); ^a^ compared to PW, ^b^ to CQ 25 mg, and ^e^ 600 mg of ML: ^∗^*P* < 0.05, ^∗∗^*P* < 0.01, ^∗∗∗^*P* < 0.001; PW: pure water (negative control); ML: crude extract of *M. lanceolata*; CQ: chloroquine. D3: values (pretreatment) on day 4; D7: values (posttreatment) on day 8.

**Table 7:** Hematocrit of malaria-infected study mice treated with *M. lanceolata* 80% methanol leaf extract in the curative test.

Test substances	Dose/day	Packed cell volume		
		D3	D7	% change
PW	10 ml/kg	44.50 ± 1.41	37.79 ± 1.32	−15.08

ML	200 mg/kg	46.17 ± 2.47	41.57 ± 1.35	−9.45^b^^∗^
	400 mg/kg	54.50 ± 0.96	50.15 ± 1.28	−7.98^a^^∗^
	600 mg/kg	45.80 ± 1.47	43.80 ± 1.29	−4.27^a^^∗∗∗^

CQ	25 mg/kg	47.62 ± 0.67	46.33 ± 0.85	−2.72^a^^∗∗∗^

Values are designated as mean ± SEM (*n* = 6); ^a^ compared to PW, ^b^ to CQ 25 mg,: ^∗^*P* < 0.05, ^∗∗∗^*P* < 0.001; PW: pure water (negative control); ML: 80% methanol extract of *M. lanceolata*; CQ: chloroquine; D3: values (pretreatment) on day 4; D7: values (posttreatment) on day 8.

**Table 8:** Weight (body) and temperature (rectal) of malaria-infected study mice treated with 80% methanol extract of *M. lanceolata* leaves in the chemoprophylactic test.

Test substances	Dose/day	Body weight (g)			Rectal temperature (°C)		
		D4	D7	% change	D4	D7	% change

PW	10 ml/kg	29.70 ± 0.94	27.60 ± 1.05	−7.12	37.25 ± 0.21	35.30 ± 0.29	−5.22

ML	200 mg/kg	28.85 ± 0.97	27.68 ± 0.87	−4.00	36.75 ± 0.14	35.40 ± 0.34	−3.68^b^^∗∗^
	400 mg/kg	32.10 ± 0.64	31.13 ± 0.84	−3.04	37.32 ± 0.29	36.33 ± 0.29	−2.63^a^^∗^^b^^∗^
	600 mg/kg	30.05 ± 0.77	29.40 ± 0.79	−2.14^a^∗^^	36.57 ± 0.29	35.93 ± 0.22	−1.72^a^^∗∗^

CQ	25 mg/kg	30.73 ± 0.62	30.90 ± 0.36	0.66^a^∗∗^^	37.28 ± 0.15	37.25 ± 0.16	−0.09^a^^∗∗∗^

Values are designated as mean ± SEM (*n* = 6); ^a^ compared to PW, ^b^ to CQ 25 mg,: ^∗^*P* < 0.05, ^∗∗^*P* < 0.01, ^∗∗∗^*P* < 0.001; PW: pure water (negative control); ML: 80% methanol extract of *M. lanceolata*; CQ: chloroquine. D4: preinfection values on day 5; D7: postinfection values on day 8.

**Table 9:** Hematocrit of malaria-infected study mice treated with *M. lanceolata* methanolic (80%) leaf extract in the chemoprophylactic test.

Test substances	Dose/day	Packed cell volume		
		D4	D7	% change
PW	10 ml/kg	50.10 ± 0.77	46.67 ± 1.17	−6.86

ML	200 mg/kg	57.63 ± 1.04	54.73 ± 1.15	−5.04^b^^∗^
	400 mg/kg	49.24 ± 1.20	47.20 ± 1.17	−4.07
	600 mg/kg	53.00 ± 0.82	51.50 ± 1.02	−2.86

CQ	25 mg/kg	47.91 ± 0.98	48.14 ± 1.32	0.41^a^^∗∗^

Values are designated as mean ± SEM (*n* = 6); ^a^ compared to PW, ^b^ to CQ 25 mg,: ^∗^*P* < 0.05, ^∗∗^*P* < 0.01; PW: pure water (negative control); ML: 80% methanol extract of *M. lanceolata*; CQ: chloroquine; D4: preinfection values on day 5; D7: postinfection values on day 8.

## Data Availability

Almost all data and materials used in the experiment are incorporated in this article.
